# Correlation Analysis of Gene and Radiomic Features in Colorectal Cancer Liver Metastases

**DOI:** 10.1155/2022/8559011

**Published:** 2022-12-24

**Authors:** Xuehu Wang, Nie Li, Haifeng Guo, Xiaoping Yin, Yongchang Zheng

**Affiliations:** ^1^College of Electronic and Information Engineering, Hebei University, Baoding 071002, China; ^2^Research Center of Machine Vision Engineering & Technology of Hebei Province, Baoding 071002, China; ^3^Key Laboratory of Digital Medical Engineering of Hebei Province, Baoding 071002, China; ^4^Affiliated Hospital of Hebei University, Baoding 071000, China; ^5^Department of Liver Surgery, Peking Union Medical College Hospital, Chinese Academy of Medical Sciences and Peking Union Medical College (CAMS&PUMC), Beijing 100730, China

## Abstract

Colorectal cancer liver metastasis (CRLM) was one of the cancers with high mortality. Clinically, the target point was determined by invasive detection, which increased the suffering of patients and the cost of treatment. If the target point was found through the relationship between early radiomic information and genetic information, it was expected to assist doctors in diagnosing disease, formulating treatment plans, and reducing the pain and burden of patients. In this study, gene coexpression analysis and hub gene mining were first performed on the gene data; secondly, quantitative radiomic features were extracted from CT-enhanced radiomic data to obtain features highly correlated with CRLM; and finally, we analyzed the relationship between gene features and radiomic feature correlations by establishing a link between early radiomic features and gene sequencing and finding highly correlated expressions. This experiment demonstrated that radiomic features could be used to mine gene attributes. Based on the four previously identified genes (NRAS, KRAS, BRAF, and PIK3CA), we identified two novel genes, MAPK1 and STAT1, highly associated with CRLM. There were specific correlations between these 6 genes and radiomic features (shape_elongation, glcm, glszm, firstorder_10percentile, gradient, exponent_firstorder_Range, and gradient_glszm_SmallAreaLowGrayLevel). Therefore, this paper established the correlation between radiomic features and genes, and through radiomic features, we could find the genes associated with them, which was expected to achieve noninvasive prediction of liver metastasis.

## 1. Introduction

Colorectal cancer (CRC) was one of the most common cancers in gastrointestinal disease [1]. According to the 2020 global cancer data, colorectal cancer ranks third among all cancers in incidence and has a high mortality rate [1]. According to study data, by 2030, there will be more than 2.2 million new colon cancer cases in the world [2], including more than 1.1 million deaths [1]. Some medical researches showed that the liver was the most prone organ for colorectal cancer to produce hematogenous metastasis [3, 4]. Therefore, the discovery of CRLM biomarkers was useful for the diagnosis and targeting of anticancer drugs.

Weighted gene coexpression network analysis (WGCNA) was a systems biology method. WGCNA can be constructed based on the expression of genes and further divided into coexpression modules [5, 6]. It can reveal the interaction mechanism of genes related to CRLM, identify potential biomarkers, and be used to identify hub genes or therapeutic targets in many fields, including mice, human brains, and various cancers. In recent years, there have been some studies on the identification of colorectal cancer coexpressed gene modules and hub genes through WGCNA [6]. For example, Shoji et al. used immunohistochemical analysis to show that ZFP57 was overexpressed in several cancers, including pancreatic cancer and colorectal cancer. The study results indicate that ZFP57 was involved in hematogenous liver metastasis [7, 8]. Wang et al. after coculturing liver metastasis cells with macrophages, the serum levels of exosomes miR-25-3p and miR-425-5p were identified through serum levels, which were related to CRLM [9]. Sclafani et al. used the mutation status of five genes (KRAS, NRAS, BRAF, PIK3CA, and TP53) to analyze the clinical features and treatment results of rectal cancer and found that mutations in these five genes would increase the recurrence and prevalence of rectal cancer [10].

Radiomics was a new and effective quantitative analysis method based on high-throughput features of medical radiomics [11–13], including five steps: image acquisition, image segmentation, feature extraction, feature selection, and modeling. Through radiomic analysis, researchers can obtain information such as tumor biology, morphology, and texture [14], which can provide valuable information for disease diagnosis and prognosis and provide clinicians with professional, reliable [15, 16], and effective pathological information. Research has found that radiomics can be used to find biomarkers related to CRLM. For example, Acharya et al. studied the CT of texture characteristics with CRLM patients. Through analysis, they found that the entropy value of the liver metastasis group and normal colon tissue was different [17], and the uniformity of extrahepatic disease was also different [18]. Dohan et al. used CT portal vein images of 230 patients with colorectal cancer and liver metastases before and after chemotherapy to analyze the texture using radiomic characteristics and then establish a prediction model. Through the experimental results, they found that the radiomic characteristics were relative to the evaluation criteria of solid tumors, and RECIST 1.1 has limited potential to assess tumor response to targeted therapies. Radiographic analysis quantifies changes in tumor heterogeneity with greater accuracy than the radiologist's naked eye [19].

The current clinical methods for diagnosing CRLM mainly include abdominal enhanced CT, serum CEA, pathological staging, and liver MRI examinations [9, 20–24]. Because there were certain differences in each person's genes, doctors needed to analyze and detect whether there were mutations in their patients' related oncogenes and then conduct personalized targeted therapy.

Radiogenomics was an emerging research method for mining the correlation between radiomics and genes. By combining radiomic features such as lesion size, grayscale, and texture features with gene expression, the potential connection between the two was explored [25]. Reflect the information between multimodal radiomic characteristics and gene expression, assisting clinicians in the diagnosis of disease. For example, Segal et al. [26] used groundbreaking early research to screen out the radiomic features of 28 patients with hepatocellular carcinoma; they screened the transcriptome information of hepatocellular carcinoma and found that their radiomic features were related to gene expression. Inspired by predecessors, we found that radiogenomics had far-reaching significance for the study of CRLM.

Since there were few studies on radiogenomics for CRLM at present, we integrated disease imaging data and genomic data, extracting key features and mining potential links between the two, and then discovered genes that could reflect gene polymorphism or expression information. Radiomic features enabled more accurate image-based noninvasive disease diagnosis methods, so as to accurately select the most suitable adjuvant immunotherapy plan according to the individual situation of the patient, reducing ineffective treatment and unnecessary suffering [26].

**Figure 1:**
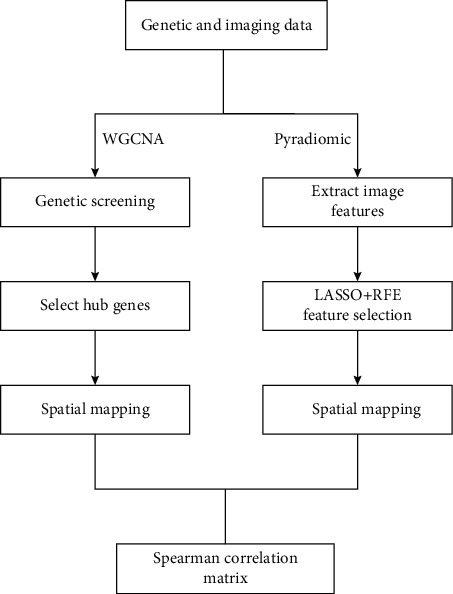
The experimental flowchart.

In this study, gene expression data were correlated with traits, and genes that were only associated with CRLM were searched to elucidate the biological significance of their genes. Then, tumor contours and feature extraction were performed on the CT-enhanced radiomics. Finally, correlation analysis was used to mine associations between gene expression and radiomic features. This method was expected to predict the relevant properties of genes from the preinvasive imaging results and then find the target points to assist doctors in developing treatment plans; the experimental flow chart is shown in Figure 1.

### 1.1. Contribution

This study was to establish the relationship between radiomic features and gene features for mining the target points of CRLM. Even if the ability was limited, this study was still expected to mine the relationship between radiomics and genes. The contributions of this research were as follows:

- Based on the gene data in this paper, this study constructed a gene coexpression network to mine the hub genes that were highly related to the disease
- Using the radiomic feature screening method combined with LASSO+RFE, the selected feature *P* value was less than 0.05, which was statistically significant and indicated that these features had a good distinguish ability for CRLM
- The Spearman matrix was established, and it was found that there was a certain correlation between radiomics and genes, which was expected to achieve a noninvasive diagnosis of CRLM target points

## 2. Method

### 2.1. Data and Preprocessing

This experiment contains 85 cases of CT and genetic data, of which 54 cases of genetic and CT radiomic data were obtained from The Cancer Imaging Archive (TCIA, https://tcia.at) and The Cancer Genome Atlas (TCGA, https://www.cancer.gov), respectively, and 31 cases of CT radiomic and genetic data were from the Affiliated Hospital of Hebei University. The criteria for patients included in this experiment were (1) adults (age ≥ 18 years); (2) the CT radiomics was clear and the location of the tumor was easy to analyze; and (3) no other cancer and family genetic history. The exclusion criteria were (1) the patient has already suffered from serious malignant tumors and other systemic disease at the time of examination in the hospital and (2) the patients take contraindicated medications that affect the results during the examination. The specific clinical information was shown in Table 1.

During the experiment, to reduce the amount of calculation and improve the reliability of the results, the top 75% of genes with the median absolute deviation were screened, and the WGCNA was used for gene coexpression clustering module analysis, and 44 gene modules were clustered. At the same time, 85 cases of radiomic data were preprocessed by normalization, and 2078 radiomic features were filtered by PyRadiomics, which reduced the amount of calculation for subsequent experiments.

### 2.2. Construction of Gene Coexpression Network and Mining of Potential Hub Genes

WGCNA was a method of studying gene set expression [9]. Use WGCNA to construct a network in which genes were regarded as points and the relationship between genes as a line [6]. Calculate the correlation based on gene expression by the Pearson coefficient and then weight the entire network to bring it close to the scale-free network distribution. Adopt a dynamic branch cutting method to divide the entire network into multiple collaborative expression modules [9]. The WGCNA package was used to perform network construction at each stage of the abovementioned acquisition. The network construction steps mainly include correlation matrix calculation, soft threshold selection, the adjacency matrix calculation, topology matrix calculation, dynamic branch cutting, module merging, and character association analysis. The adjacency matrix (*a*ij) was required to determine when constructing the network, which was asymmetric *n*∗*n* matrix with a value range of [0, 1], and its components represent the strength of the network connection at nodes. To better calculate the adjacency matrix, an intermediate variable *s*ij (coexpressing similarity) was necessary to represent the absolute value of the correlation coefficient between nodes *i* and *j*, which were defined as follows [6]:

(1) $$\begin{array}{c} s_{\text{ij}}=\left|\text{cor}\left(x_{i},x_{j}\right)\right|. \end{array}$$

When *i* ≠ *j* in the formula, it represents two different gene modules, and the weighted coexpression network can also be characterized by improving the similarity between coexpression and power, as shown in the formula (2)

(2) $$\begin{array}{c} {\text{a}}_{\text{ij}}={\text{s}}_{\text{ij}}^{\beta }. \end{array}$$

Screening out highly correlated gene modules can better reflect the overall gene expression and explain the interaction mechanism between genes. Use the functional enrichment method (GO analysis) to compare genes or genomes with functional databases, perform overexpression analysis and functional annotation, and provide references for the study of gene molecular mechanisms of CRLM [27].

The hub gene was a gene that plays an important role in the network and can represent the genetic characteristics of the module to a certain extent. The preliminary experiment was to determine the modules that were significantly related to the clinical features to be studied. Gene significance (GS) and module membership (MM) were used to screen core genes. GS described the correlation between genes and clinical traits, which reflected the relationship between genes and traits, and MM described the correlation between genes and modular vectors, which gets the core position of the gene in the module [10]. In this experiment, the GS value and MM value of the gene were calculated (it can also be expressed by the *K* value). The condition for screening the core gene was to satisfy both |GS| > 0.1 and |MM| > 0.8 [2].

(3) $$\begin{array}{c} {\text{GS}}_{i}=-\log P_{i},\\ K_{\text{cor},i}^{\left(q\right)}≔\text{cor}\left(x_{i},E^{\left(q\right)}\right). \end{array}$$

In the formula, *P*_*i*_ represents the significance of the difference between genes, and *E*^(*q*)^ represents the characteristic gene of module *q*.

Individual genes cannot fully function, and they need to coordinate with each other to function. Therefore, this experiment uses the MCODE method to study the protein-protein interaction information of the significantly related hub genes and gene modules and determine the hub nodes of the subnetwork according to the node degree and similarity center. Only by finding the hub genes related to CRLM through genetic analysis could we make more targeted connections with radiomics and discover potential links between noninvasive diagnosis and gene features.

### 2.3. Radiomic Feature Extraction

#### 2.3.1. Image Preprocessing and Lesion Segmentation

We use GE Discovery HD750 64-Slice CT scanner. Scanning method: patients need to fast for 6-8 hours before performing abdominal plain scan and enhanced scan. Scanning parameters include layer thickness 5 mm, pitch 0.992, scanning field of view 350 mm × 350 mm, matrix 512 × 512, tube voltage 100~120 kV, and tube current 160~300 mA. The contrast agent was injected through the cubital vein with a flow rate of 3.0~3.5 ml/s and a dose of 0.5 ml/kg. The acquisition time of the liver's arterial phase, portal vein phase, and delayed phase scan images was 30-35 s, 50-60s, and 180 s after the injection of the contrast agent. Taking into account that the lesions were displayed most clearly in the portal vein phase, in order to avoid errors, the portal vein phase CT-enhanced images will be selected for radiomic analysis.

Segmentation of the region of interest (ROI) was the basis for feature extraction and prediction model establishment in radiomics. In this experiment, a radiologist with 5 years of work experience performed the outline of the lesion area of the internal 31 cases of CT-enhanced radiomic data set, keeping a distance of about 2-3 mm from the edge of the tumor. In order to ensure the accuracy of the outline results, another senior radiologist with 10 years of working experience checks and outlines the results.

#### 2.3.2. Radiomic Feature Extraction and Selection

This experiment uses the PyRadiomics to extract radiomic features of the outlined lesions, including the first-order statistics, the shape elongation, the first-order exponential characteristics, the informational measure of correlation, the gray-level size zone matrix, and radiomic features after wavelet, square, logarithmic, and other filters.

When doing the radiomics part of the experiment, the patients have been divided into two groups, of which the test group data represents 70% of all the data, and the verification group data represents 30% of all the data.

In feature selection, to make the data more readable, this experiment tried to use recursive feature elimination (RFE) [28], analysis of variance (ANOVA) [29], the least absolute shrinkage and selection operator (LASSO) [30], and other algorithms for feature selection [25]. It can effectively avoid the phenomenon of experimental overfitting and screen out the radiomic features that could better identify the disease.

### 2.4. Correlation Analysis of Radiomic Features and Gene Features

**Figure 2:**
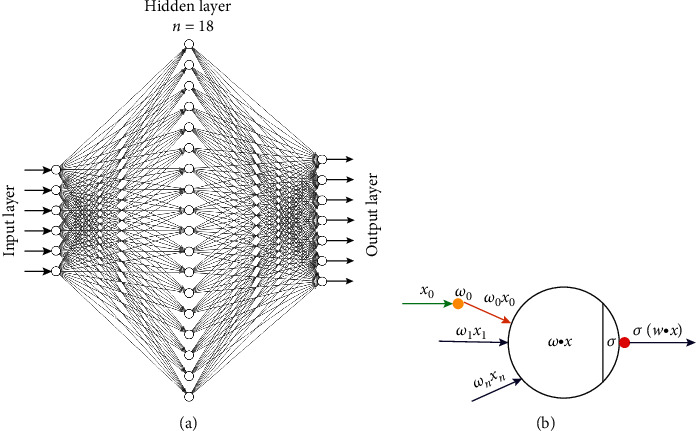
(a) The overall structure of the neural network. (b) The process of neuron operation.

Inspired by the biological nervous system and the above experiments, this study introduced the neural network method to establish the connection between the radiomic feature and the genetic feature. The learning framework for this article was PyTorch, which was mainly composed of an input layer, a hidden layer, and an output layer. The hidden layer uses the output of the previous layer as the input of this layer to iteratively update the parameters. In this network, the input layer has 6 neurons as input signals, and these input signals were transmitted through weighted connections; the total input value received by the neuron will be compared with the neuron threshold and then subjected to linear function operations. Finally, the output of 7 neurons was produced. The overall framework is shown in Figure 2(a), and the calculation process between each neuron is shown in Figure 2(b). Through continuous experiments, it was found that when the number of neurons in the hidden layer was 18, the output of the network was the best, so the number of neurons in the hidden layer in this experiment was 18. By constructing a neural network, the data in two different spaces was mapped to the same space to study the correlation between radiomic features and gene features.

In order to enhance the consistency of the experiment, a specific Spearman correlation analysis was performed on the genetic data and radiomic data in the same space obtained from the above experiment, and a relationship between the two was found.

### 2.5. Statistical Analysis

This study uses the Spearman matrix to find the potential connection between radiomic and gene features and calculate the Pearson correlation factor to find the correlation between gene expressions. Python (version 3.7) and R (version 4.0.4) ggplot package were plotted. *P* < 0.05 was regarded as statistically significant.

## 3. Results

### 3.1. Construction of Weighted Gene Coexpression Network

First, this experiment performs a weighted gene coexpression network analysis on the public data set and the internal private gene set and then constructs an adjacency matrix for the preprocessed and screened genes, using WGCNA for cluster analysis to cluster genes with similar gene expressions.

**Figure 3:**
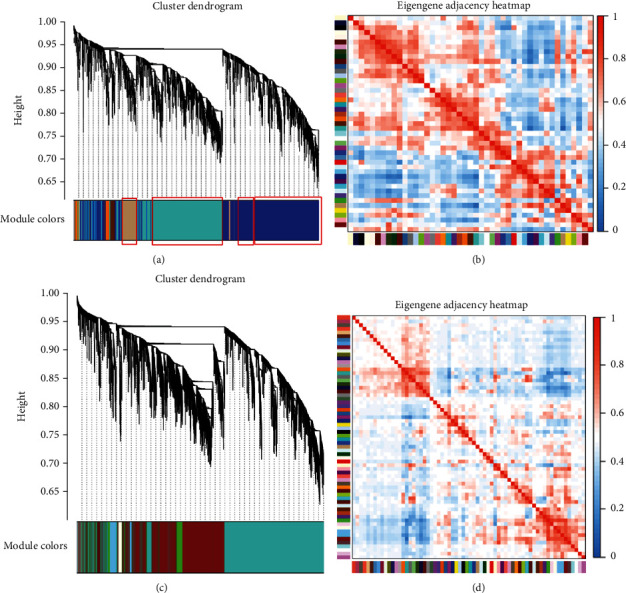
(a) and (c) were gene module clustering. (b) and (d) were gene module associative heat map.

**Figure 4:**
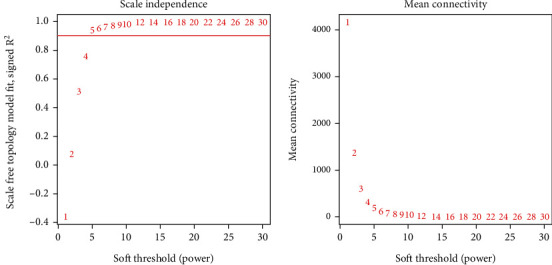
Module soft threshold selection.

Using the general parameters of network construction, the data of this study were networked to find that the correlation between clustering results and gene heat maps was poor, as shown in Figures 3(c) and 3(d). It could be found that most of the genes exist in the blue and midnight blue modules, as shown in Figures 3(a) and 3(b). Therefore, through targeted analysis of the data set used in this study, it was found that when our network soft threshold *β* was set to 5, the module's mean connectivity was the best and had a normal distribution, and the gene clustering effect and gene heat map results were the best, as shown in Figure 4.

Secondly, according to the gene modules clustered, the eigengenes were extracted to calculate the adjacency between the modules, and the heatmap was used for visualization, as shown in Figure 3(b). It can be seen from the figure that 44 gene modules were divided into two regions: the upper-left corner and the lower-right corner. The internal adjacency was relatively high, indicating that the degree of correlation between gene modules was relatively good, which was of research significance.

**Figure 5:**
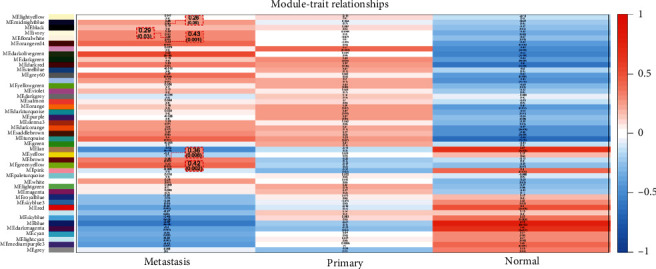
The traits of the eigengene.

Finally, the association between gene modules and clinical characteristics was mined, and the results are shown in Figure 5. The CRLM was on the left side, primary colorectal cancer was in the middle, and the normal colon was on the right side. It could be seen from the experimental results that the positive correlation module of the normal colon was negatively correlated with the other two states. In order to improve the feasibility of the experiment, select 5 gene modules with the highest correlation with the positive correlation positive liver metastasis.

#### 3.1.1. High Adjacency Subnetwork Data Mining

**Figure 6:**
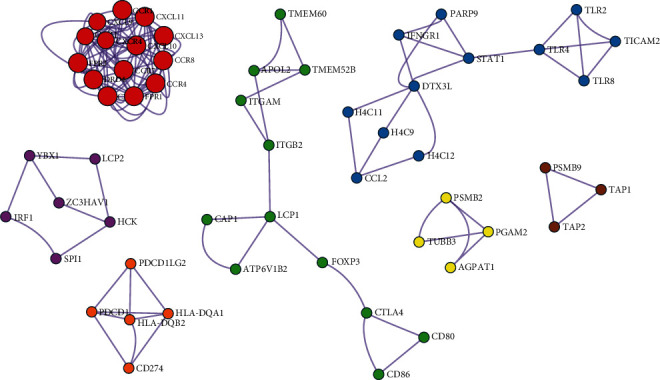
High adjacency subnetworks mined with MCODE.

The genes with low relevance were eliminated, incorporating the remaining 617 genes for subsequent experiments. The experiments mine the high adjacent subnetwork from the gene coexpression networks through MCODE analysis (http://metascape.org) and annotate these subnetworks (Figure 6 and Table 2 shown). The functional annotations of these subnetworks include peptide ligand-binding receptors, G*α* signaling events, positive regulation of nitric-oxide synthase biosynthetic process, DTX3L-PARP9-STAT1 complex, immune response-activating signal transduction, immune response-regulating signaling pathway, and integrin MAPK1 signal transduction connection, of which STAT1 and MAPK1 were highly related to cancer, and others were related to metabolism and microenvironment, as shown in Figure 6.

STAT1 mainly promotes cell apoptosis, inhibits cell proliferation, and negatively regulates the cell cycle. Some studies had found that it promotes tumor cell proliferation and drug resistance [31, 32]. The MAPK1 gene plays an important role in regulating the differentiation and growth of cells. According to the mechanism of the signaling pathway, as long as there were protein functional problems in the signaling pathway, it will cause serious disease, and this disease was generally related to tumors [33].

### 3.2. Radiomic Feature Extraction and Screening

#### 3.2.1. Lesion Outline and Radiomic Feature Extraction

**Figure 7:**
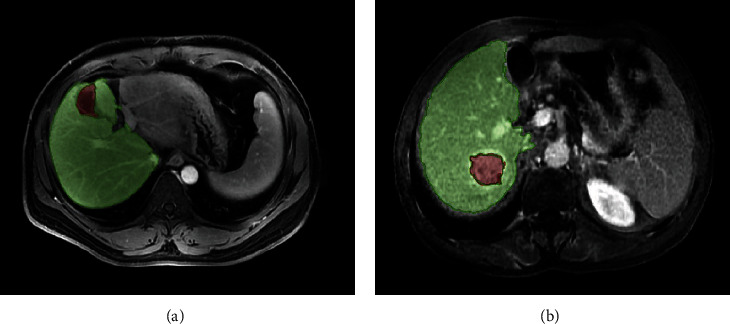
Results of lesion delineation. The green area was normal tissue, and the red area was the tumor outlined.

Segmentation of ROI was a key step for radiomic feature extraction. In this experiment, the lesion was outlined on 85 cases of CT-enhanced radiomic data inside, and the MITK tool was used to draw the outline of the lesion area. The results of the lesion delineation are shown in Figure 7. Radiomic feature extraction was performed on the original CT image and the corresponding mask radiomic, and a total of 2,078 features were extracted, including shape features, first-order statistical features, and second-order texture features.

#### 3.2.2. Radiomic Feature Screening

**Figure 8:**
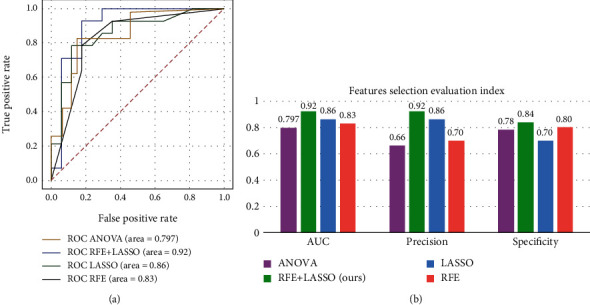
Result comparison chart. (a) The comparison chart of ROC results of feature selection methods and (b) the comparison chart of AUC, precision, and specificity results.

In order to increase the desirability of the data, filters were used to further filter the features; among which, wavelet features and Laplacian Gaussian filter features were the main features. The experiments used ANOVA, LASSO, RFE, and other algorithms for feature selection of the data, and the results are shown in Figure 8. Four feature selection methods, ANOVA, LASSO, RFE, and LASSO+RFE, were tested. After comparison, it was found that the AUC, accuracy, and specificity of the ANOVA method were 0.797, 0.66, and 0.78; the AUC, accuracy, and specificity of the LASSO method were 0.86, 0.86, and 0.84; and the AUC, accuracy, and specificity of the RFE method were 0.83, 0.70, and 0.80. However, the LASSO+RFE feature selection method proposed in this study is the most effective, with AUC, accuracy, and specificity of 0.92, 0.92, and 0.84.

**Figure 9:**
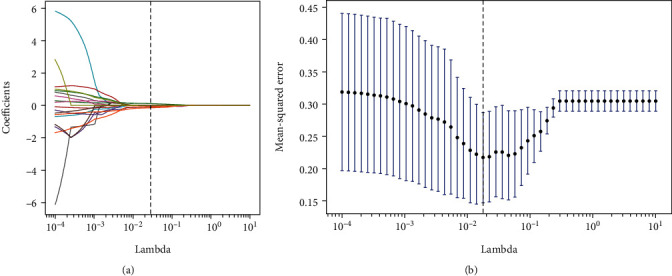
Determination of feature screening parameters. (a) Determination of the best parameter lambda. (b) The best variable coefficient of the LASSO regression model.

Figure 9(a) is the preliminary feature screening using tenfold cross-validation. When the parameter curve approaches a straight line, the mean square error between features was the smallest, and the lambda feature parameter has the best effect; Figure 9(b) is the change of feature coefficient change trajectory; when the trajectory approaches 0, the best control parameters of the LASSO model can be obtained, and finally, seven radiomic features related to CRLM were screened out, namely, glszm_SmallAreaLowGrayLevelEmphasis (glszm_SALGLE), glsm_Imc2, gradient_glszm_SizeZoneNo-nUniformity (g_glszm_SZNUN), Shape_Elongation, firstorder_Range, exponent_firstorderRange (e_firstorder_Range), and gradient_glszm_SmallAreaLowGrayLevel Emphasis (g_glszm_SALGLE), as shown in Table 3. To investigate the statistical significance of the proposed method over compared methods on each metrics, the Wilcoxon signed-rank test was employed to conduct the statistical analyses.

### 3.3. Correlation Analysis of Radiomic Features and Gene Features

**Figure 10:**
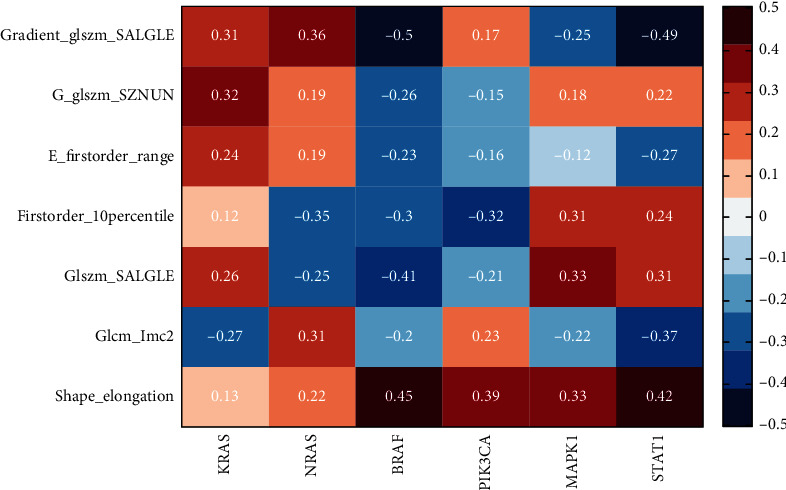
Correlation analysis between radiomic features and genetic features. The radiomic features were indicated by the initial letter, the horizontal axis was the radiomic features, and the vertical axis was the gene feature. The stronger the correlation, the darker the color.

Map the radiomic features and gene feature data in the same space through the neural network; neural network setting parameters are shown in Table 4, and use the combination with genomics and multimodal radiomics to analyze the correlation and combine the KRAS, NRAS, BRAF, and PIK3CA found in existing studies. Spearman's correlation analysis was carried out with the newly mined MAPK1 and STAT1 and 7 screened radiomic features in this experiment, and the results were visualized with heatmap. The results are shown in Figure 10.

Through the results of visualization, the correlation coefficient between MAPK1 and glszm_SALGLE and Shape_Elongation was 0.33, the correlation coefficient with firstorder_10percentle was 0.31, the correlation coefficient with g_glszm-SZNUN was 0.18, the correlation coefficient with glcm_lcm2 was -0.22, the correlation coefficient with g_glszm_SALGLE was -0.25, and the correlation coefficient with e_firstorder_Range was -0.12; the correlation coefficient between STAT1 and glszm_SALGLE was 0.31, the correlation coefficient with Shape_Elongation was 0.42, the correlation coefficient with firstorder_10percentle was 0.24, the correlation coefficient with g_glszm-SZNUN was 0.22, the correlation coefficient with glcm_lcm2 was -0.37, the correlation coefficient with g_glszm_SALGLE was -0.49, and the correlation coefficient with e_firstorder_Range was -0.27.

In conclusion, STAT1 and MAPK1 were positively correlated with the four radiomic features of Shape_Elongation, glszm_SALGLE, firstorder_10Percentile, and glszm_SZNUN, and negatively correlated with the three radiomic features of glcm_Imc2, firstorder_Range, and glszm_SALGLE. Therefore, the attributes of genes can be qualitatively predicted by the radiomic features.

## 4. Discussion

Roy et al. [34] performed radiomic analysis of triple-negative breast cancer patients and found that tumor volume, noise characteristics, and image resolution had a significant impact on radiomic analysis in a common clinical study. Forty-eight radiomic features were extracted from manually segmented 2D and 3D images, and 16 radiomic features were obtained by feature correction with clinical features, and finally, it was found that the features of grayscale travel length matrix (GLRLM) and grayscale size region matrix (GLSZM) were determined to be the most sensitive to noise. The kurtosis and travel variance (RLV) of the radiomic features of GLSZM were found to be the most sensitive to the resolution variation of T1w and T2w of MRI.

Mokrane et al. [35] extracted 12 sets of 1160 quantitative features from CT radiomics and used machine learning techniques to classify liver nodules into hepatocellular carcinoma and nonhepatocellular carcinoma, resulting in a validation set ROC of 0.66. The results showed that the radiomic histological features had good diagnostic power for hepatocellular carcinoma in patients with cirrhosis with indeterminate liver nodules.

Roy et al. [36] used radiomic features to screen 64 features from 131 clinical features to consistently predict treatment response in patients with triple-negative breast cancer. Classification and regression tree (CART), Naïve Bayes (NB), and support vector machine (SVM) were used for feature selection, which ultimately yielded a prediction accuracy of 77%. Meanwhile, the scholar proposed [37] an automation-based brain binarization MRI method for brain disease detection and feature extraction in preprocessing, which effectively improves the preprocessing method and reduces the error. This proposed method effectively solves the brain imaging MR binarization problem. González-Castro et al. [38] extracted texture features of CT radiomics of colorectal cancer patients and used support vector machines and random forest models for classification studies with 83% classification accuracy, and the experimental results demonstrated that texture feature analysis can quantitatively assess tumor heterogeneity by analyzing the distribution and relationships of pixels in images.

With the continuous advancement of modern technology, radiomics has been widely studied in medicine [39]. For example, Jia et al. studied the correlation between the radiomic features of lung cancer and its genetic features [40]. The experiment found that EGFR gene mutations are crucial in the treatment of lung cancer through noninvasive radiomic features [40]. Zhu et al. found that the gene transcription activity of breast cancer was positively correlated with tumor size, shape, and blurred edges. This study provides a basis for imaging technology as a noninvasive detection of cancer genes [41].

The above studies were radiogenomic studies of different disease; however, there were few studies for CRLM, and clinical treatment for CRLM usually involved genetic sequencing of patients' pathological tissue sections, which was an invasive test that increased patients' pain and economic burden. Therefore, studies related to the use of radiomics to predict genetic attributes were valuable, and by integrating radiomic information with genomic information, it was expected to achieve adjuvant treatment options for patients with CRLM, reducing patient pain and improving patient survival.

When radiomic features were selected, it was found that the commonly screened features were not sufficiently targeted for the data; so by interfeature characteristics, this study compared the common feature selection methods with the combined LASSO+RFE method used in this study and found that the AUC results of the feature selection method used in this experiment were better. The useful features selected by this method adequately consider the correlation between features and targets and the redundancy between features. When establishing the connection between the gene and radiomic features, this study encountered that the data of both gene and radiomics were not under the unified spatial dimension, and there would be some errors if this study performed correlation analysis directly, so this experiment used a neural network to operate on the unified spatial dimension for both data and mapped them under the unified spatial dimension to enhance the readability of the data.

The advantages of this article were to predict gene attributes using radiomic features and then determine the target point; the second was to use neural network research methods to map the two types of data in a unified space through certain neuron operations to enhance the readability and interpretability of the data. However, our research also has some limitations. Due to the small number of samples of gene expression and CT radiomics, this may limit the effectiveness of the display of related information between genes and radiomics. Therefore, future research should increase the number of samples and improve feature extraction algorithms to find more radiomic features and establish models for early prediction of multiple diseases and then develop recurrence monitoring strategies for patients.

## 5. Conclusion

In this study, this study first clustered multiple gene modules using WGCNA to mine hub genes that were highly correlated with CRLM. Secondly, radiomic features were extracted from CT-enhanced data, and feature selection was performed by a combined LASSO and RFE feature selection method to screen out radiomic features with better discrimination. Since the two types of features were not in the same space, a neural network was used to map the two sets of features into the same space. Finally, Spearman's correlation analysis was performed to find the radiomic features that were correlated with the gene modules. The experimental results showed that there was indeed a significant correlation between radiomic features and gene features. This study was expected to provide help for the noninvasive prediction of CRLM disease.

## Figures and Tables

**Table 1:** Patient specific clinical information.

Clinical data	Quantity	Percentage
Gender male/female	19/12	61.29%/41.94%
Average age male/female	53/58.78
Whether there was lymph node metastasis around yes/no	22/9	70.97%/29.03%
Primary disease site-right colon	6	19.36%
Primary disease site-left colon	2	6.45%
Primary disease site-part of the sigmoid colon and rectum	7	22.58%
Primary disease site-sigmoid colon	4	12.9%
Primary site-rectum	12	38.71%
CEA value >5/<5	21/10	67.74%/32.26%
CA724 value >6/<6	11/20	35.48%/64.52%

**Table 2:** Function enrichment of subnetworks identified by MCODE.

Subnet number	Function annotation number	Function annotation description	Log10(*P*)
MCODE_1	R-HSA-373076	Class A/1 (rhodopsin-like receptors)	-27.0
MCODE_1	R-HSA-375276	Peptide ligand-binding receptors	-26.9
MCODE_1	R-HSA-418594	G alpha (i) signaling events	-25.8
MCODE_2	GO:0051770	Positive regulation of nitric-oxide synthase biosynthetic process	-10.5
MCODE_2	CORUM:7385	DTX3L-PARP9-STAT1 complex	-10.2
MCODE_2	GO:0071346	Cellular response to interferon-gamma	-10.2
MCODE_3	Ko05323	Rheumatoid arthritis	-9.6
MCODE_3	Hsa05323	Rheumatoid arthritis	-9.4
MCODE_3	ko04514	Cell adhesion molecules (CAMs)	-8.6
MCODE_4	GO:0002757	Immune response-activating signal transduction	-3.6
MCODE_4	GO:0002764	Immune response-regulating signaling pathway	-3.5
MCODE_4	GO:0002253	Activation of immune response	-3.5
MCODE_5	R-HSA-389948	PD-1 signaling	-15.6
MCODE_5	R-HSA-388841	Costimulation by the CD28 family	-13.1
MCODE_5	Hsa04514	Cell adhesion molecules	-11.4
MCODE_7	GO:0002479	Hallmark coagulation	-7.7
MCODE_7	GO:0042590	Complement cascade	-7.6
MCODE_7	R-HSA-1236974	ER-phagosome pathway	-7.6

**Table 3:** Feature selection analysis (Wilcoxon's tests were used in this experiment, and *P* < 0.05 represented statistical significance).

Feature name	*P* value
glszm_SALGLE	<0.01
Shape_Elongation	<0.05
firstorder_10percentle	<0.02
g_glszm-SZNUN	<0.01
glcm_lcm2	<0.01
g_glszm_SALGLE	<0.01
e_firstorder_Range	<0.01

**Table 4:** Neural network setting parameters.

Parameters	Values
Number of hidden layers	18
Optimizer	Adam
Learning rate momentum	0.0001
Momentum	0.9
Dropout	0.35

## Data Availability

The study data used to support the findings of this study are available from the corresponding author upon request.
